# Varied human milk oligosaccharides in human milk from mothers treated with antidepressants and anti-inflammatories

**DOI:** 10.1038/s41390-025-04650-5

**Published:** 2025-12-16

**Authors:** Essi Whaites Heinonen, Gretchen Bandoli, Bianca Robertson, Chloe Yonemitsu, Lars Bode, Hannah Riedy, Kerri Bertrand, Christina Chambers

**Affiliations:** 1 Department of Pediatrics, University of California San Diego, La Jolla, CA, USA.; 2 Division for Pediatrics, Department of Clinical Science, Intervention and Technology, Karolinska Institutet, Stockholm, Sweden.; 3 Human Milk Institute (HMI), University of California San Diego, La Jolla, CA, USA.; 4 Larsson-Rosenquist Foundation Mother-Milk-Infant Center of Research Excellence (LRF MOMI CORE), University of California San Diego, La Jolla, CA, USA.; 5 Department of Pediatrics, School of Medicine, Duke University, Durham, NC, USA.

## Abstract

**BACKGROUND:**

Human milk oligosaccharides (HMOs) are abundant carbohydrates in human milk, providing infants with numerous health benefits, but it is not known whether maternal medications could affect them. We aimed to study the association between antidepressant and anti-inflammatory medication use and HMO composition in human milk.

**METHODS:**

This study included 544 human milk samples from the UC San Diego Human Milk Biorepository collected between October 2014 and November 2022 from participants treated with antidepressants, anti-inflammatories, or neither. Concentrations of 19 HMOs were analyzed by high-performance liquid chromatography and compared between groups stratified by secretor status.

**RESULTS:**

In secretors (*n* = 390), total HMO concentrations were significantly lower in samples exposed to antidepressants and anti-inflammatories than in unexposed samples, median (Interquartile Range [IQR]) 12.43 (5.32) and 12.71 (4.94) vs 16.10 (2.25) mmol/L, but not in non-secretors (*n* = 154). In both secretors and non-secretors, samples exposed to antidepressants had higher percentages of several fucosylated oligosaccharides than unexposed.

**CONCLUSIONS:**

The HMO composition varied between human milk samples from mothers treated with antidepressant and anti-inflammatory medications and mothers treated with neither. Whether these associations are causal or could affect breastfed infants requires further investigation. The findings do not discourage the use of these medications during lactation.

## INTRODUCTION

Human milk oligosaccharides (HMOs) are abundant carbohydrates found in human milk and comprise the third largest solid component of human milk after lactose and lipids.^1,2^ They are known to provide the breastfed infant with numerous health benefits, such as helping in forming the gut microbiome, protecting from harmful pathogens, altering immune responses, and potentially also promoting healthy brain development and affecting infant growth beyond the breastfeeding period.^1,3,4^ To our knowledge, the potential impact of maternal chronic medication use on HMO composition has not been previously studied.

HMOs are composed of different combinations and structures of the monosaccharides glucose, galactose, N-acetylglucosamine, fucose, and sialic acid, found in humans as N-acetylneuraminic acid.^5,6^ There are over 200 different HMOs recognized to date, and they are often categorized depending on if they are fucosylated, sialylated, both fucosylated and sialylated, or neither.^1,2,7^ The fucosylated HMOs, including 2’-fucosyllactose (2’FL), 3-fucosyllactose (3FL), difucosyllactose (DFLac), lacto-N-fucopentaose (LNFP) I-III, difucosyl-lacto-N-tetraose (DFLNT), fucosyl-lacto-N-hexaose (FLNH) and difucosyl-lacto-N-hexaose (DFLNH), are suggested to exhibit immunoprotective effects on the breastfed infant,^8,9^ whereas the sialylated HMOs, including 3’sialyllactose (3’SL), 6’sialyllactose (6’SL), sialyl-lacto-N-tetraose b (LST b), sialyl-lacto-N-tetraose c (LST c), disialyl-lacto-N-tetraose (DSLNT) and disialyl-lacto-N-hexaose (DSLNH), are suggested to promote infant brain development and influence the maturation of the immune system.^10–12^

Low levels of one specific sialylated HMO, DSLNT, have been found to be predictive of necrotizing enterocolitis (NEC). A threshold DSLNT value below 0.24 mmol/l has been reported to have a specificity and sensitivity of 0.9 of predicting NEC. NEC can be a devastating complication in preterm infants, with inflammation of the intestine causing bacterial invasion, leading to cellular damage and necrosis of the bowel. Thus, there may be clinical relevance for monitoring levels of DSLNT in breastmilk fed to preterm infants and finding ways of maintaining them above the threshold.^1,13–17^

Based on the activity of the secretor gene that encodes the enzyme fucosyltransferase 2 (FUT2), women can be divided into secretors and non-secretors, with 60–95% of women being secretors globally.^1,2,18^ Other factors identified to affect the HMO composition of human milk are infant age and gestational age at delivery, with the total HMO concentrations being higher in preterm than in term milk.^7,19^ Other factors associated with variations in HMO composition are maternal age, race, ethnicity, geographic location, diet, body mass index (BMI) and parity, and exclusive breastfeeding and infant growth are also suggested, but not confirmed, to affect the composition.^7,11,20–29^

Globally, over 50% of women use at least one medication in the postpartum period.^30,31^ While the primary focus of research on medication use during lactation has been on the drug transfer and the potential for direct effects on the breastfed infant, indirect effects on the milk composition have been largely unexplored. A recent study found an association between the relative abundance of several HMOs and maternal depressive symptoms and stress, and suggested this association was linked to the maternal use of antidepressant medications.^32^ To our knowledge, this has not been previously studied, and therefore we aimed to determine whether the composition of HMOs differed between human milk samples from mothers treated with antidepressants, with anti-inflammatory medications, and with neither.

## MATERIALS AND METHODS

### Subjects and Sample Collection

**Fig. 1:**
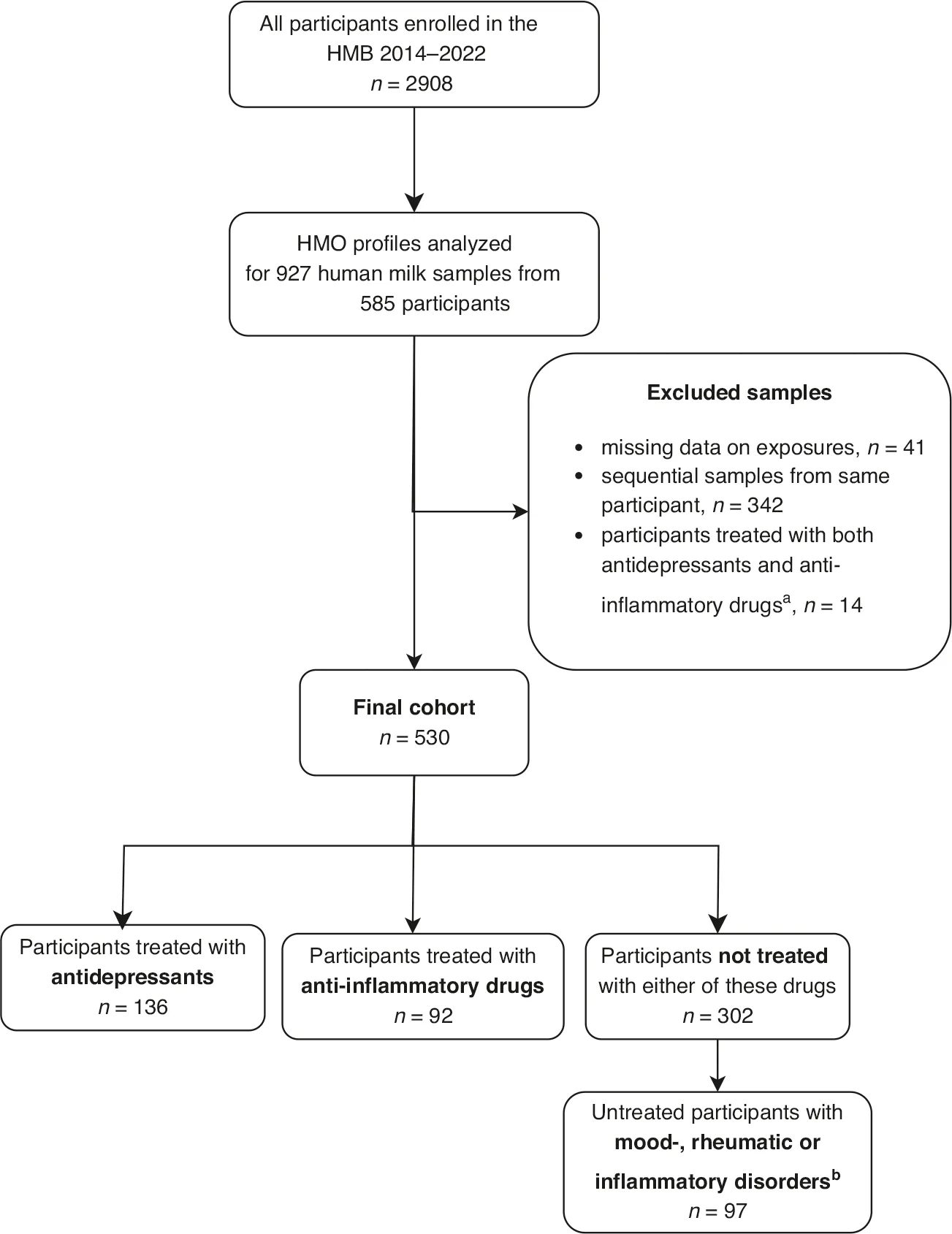
Flow-chart over the study cohort. ^a^Excluded from the main analyses but included as a separate exposure group in the analysis of disialyllacto-N-tetraose (DSLNT). ^b^Included in the main analyses in the group of non-exposed participants where maternal underlying disorders were adjusted for. Included as a separate subgroup in the analysis of DSLNT. HMO Human Milk Oligosaccharide, HMB The UC San Diego Human Milk Biorepository

This cross-sectional study was a secondary analysis of data from the UC San Diego Mommy’s Milk, Human Milk Biorepository (HMB), including samples analyzed for HMOs in June 2018 and in September 2023 (Fig. 1).^32^ Lactating volunteers aged 18 years old and older from U.S. and Canada were invited to provide milk samples to the HMB between October 2014 and November 2022. Participants provided written informed consent for the use of their milk for research and completed an interview about their sociodemographics, maternal and infant health history, lifestyle, and exposures to medications and recreational substances. Per protocol, participants were asked to collect 50 mL of milk up to 24 hours before their scheduled interview. Samples were collected off-site in participants’ homes, refrigerated at 0 to 4°C for up to 24 hours and shipped overnight on ice to the HMB Research Center, where they were aliquoted and stored at −80°C until the time of analysis.^23,33^ For this study, the first milk sample for each eligible participant was included.

### Exposures

**Fig. 2:**
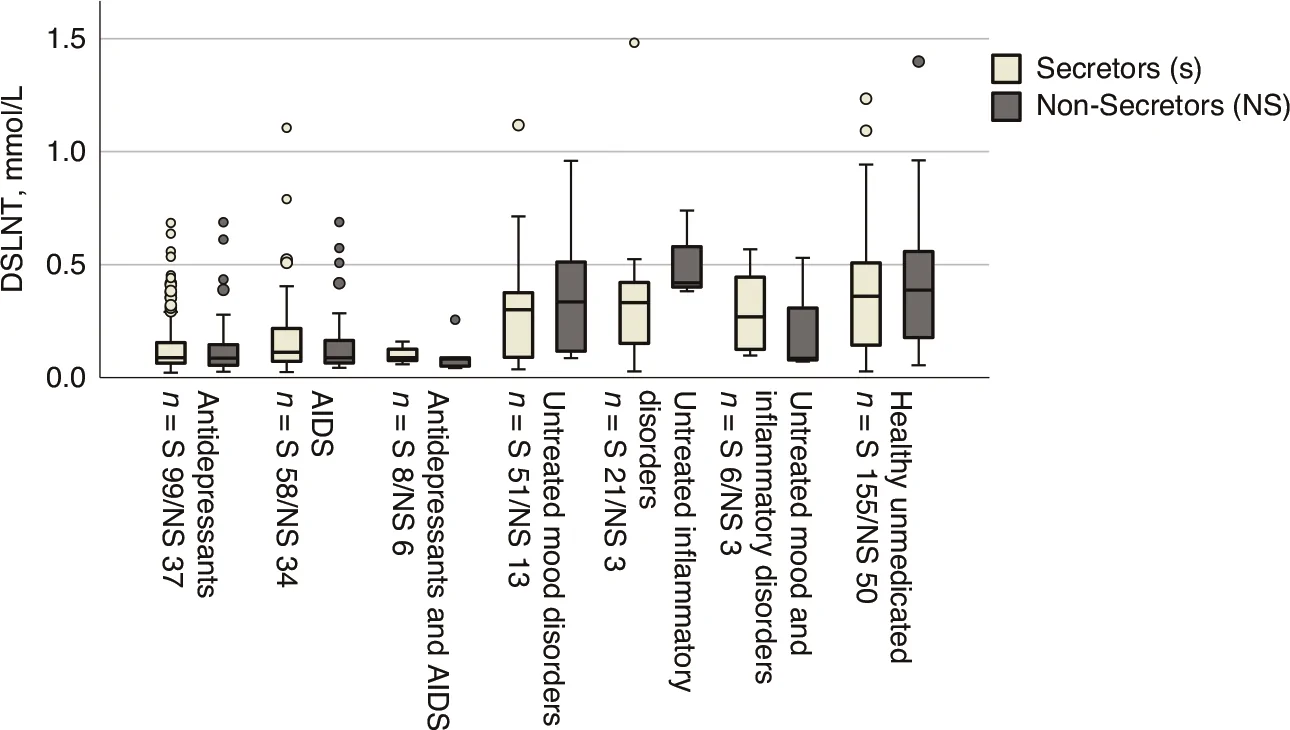
Disialyllacto-N-Tetraose (DSLNT) concentrations in human milk from participants with and without chronic medications and disorders, by secretor status. Boxplots of the median (midline of box) levels of disialyl-lacto-N-tetraose (DSLNT) measured in mmol/L with interquartile range (box), range (whiskers) and outliers (markers) in milk samples from secretors (dark) and non-secretors (light) treated with antidepressants, anti-inflammatory drugs (AIDs), antidepressants and AIDs, and healthy unmedicated participants, and participants with untreated mood and/or inflammatory disorders. DSLNT = Disialyllacto-N-Tetraose; AIDs = anti-inflammatory drugs, Secretors = S, Non-Secretors = NS

Medication exposures were captured day-by-day for the 14 days prior to sample collection, including the dose and indication for each medication. Antidepressant exposure was defined as treatment with selective serotonin reuptake inhibitors (SSRIs), selective serotonin and norepinephrine reuptake inhibitors (SNRIs), and tricyclic antidepressants (TCA), and anti-inflammatory drug exposure as treatment with monoclonal antibodies (MABs), systemic steroids or traditional disease-modifying rheumatic agents (DMARDS) within these 14 days. Participants not treated with either antidepressants or anti-inflammatory drugs in this time period were classified as unexposed, and participants treated with both antidepressants and anti-inflammatory drugs were excluded from the main analysis but included in the secondary analysis on DSLNT (Figs. 1, 2).

### Covariates

Maternal factors of interest included maternal age (in years; continuous) and body mass index (BMI) (in kg/m^2^; continuous) at the time of sample collection; race/ethnicity (non-Hispanic White, non-Hispanic Black, Hispanic, other); smoking (yes/no); and current underlying disorders. For the antidepressant model, a categorical composite variable for current underlying disorders was created from current diagnoses of psychiatric disorders and current depressive symptoms measured with the Edinburgh Postnatal Depression Scale (EPDS), with one point given for any diagnosis and one point for EPDS scores ≥ 10 points or a positive answer on question 10 on self-harm, regardless of the score.^34,35^ The models with anti-inflammatory drugs were adjusted for current maternal diagnoses of inflammatory or rheumatic disorders (yes/no). Infant factors of interest included infant age at the time of sample collection (in years; continuous); sex (male/female); and exclusive breastfeeding status (exclusive breastfeeding, breastfeeding and infant formula, breastfeeding and solid foods, all three). Sample collection-related factors included storage time of the sample (in months; continuous).

### Outcomes

HMO analyses were performed by high-performance liquid chromatography (HPLC) after fluorescent derivatisation with 2-aminobenzamide (2AB) as previously described.^36,37^ The following 19 HMOs were detected based on retention time comparison with commercial standard oligosaccharides and mass spectrometry analysis: 2’FL, 3FL, 3’SL, 6’SL, lacto-N-tetraose (LNT), lacto-N-neotetraose (LNnT), LNFP I-III, LSTb and LSTc, DFLNT, lacto-N-hexaose (LNH), FLNH, DFLac, DFLNH, fucosyl-disialyl-lacto-N-hexaose (FDSLNH), DSLNT, and DSLNH.^14^ In addition to absolute concentrations, the percentage of each HMO of the total HMO concentration was calculated and expressed as relative abundance.^14^ Maternal secretor status was defined by the presence or near absence of 2’FL of LNFP I, the two HMOs synthesized by FUT2.^14^ The HMOs were studied as both absolute concentrations (μg/L and mmol/L) and relative abundances, as well as absolute concentrations of HMO-bound sialic acid and fucose (mmol/L).

Due to the recent finding of low DSLNT levels in human milk as predictive of NEC in preterm infants, and due to the main stratified analyses showing levels of DSLNT under the threshold of risk in all exposed groups among both secretors and non-secretors, DSLNT levels were studied further in a post-hoc analysis. In this analysis, milk samples from mothers treated with both antidepressants and anti-inflammatories were studied as a third, separate, exposure group, and the unexposed samples from mothers with untreated mood and inflammatory disorders were separated from the other unexposed samples and selected to represent a disease-matched comparison group. As some studies have pointed towards higher HMO levels in preterm milk, the preterm and early milk samples were also studied separately from the samples from mothers with older infants. Early samples were defined as samples expressed before 44 weeks of gestation (i.e., up to 4 weeks after estimated due date for delivery), and late samples were after 44 weeks of gestation (i.e., more than 4 weeks after estimated due date).

### Statistical method

Background characteristics of the included mothers and infants were described as numbers and percentages and means and standard deviations (SD), and compared between the exposure groups with chi square for categorical variables and independent samples t-tests for continuous variables. Total concentrations of HMOs, HMO-bound sialic acid, and fucose, and median relative abundances of the individual HMOs were described as means and SD and medians, and inter quartile ranges (IQR) and separately compared between milk samples from participants from each exposure group with samples from those exposed to neither. All analyses were performed separately for secretors and non-secretors. Crude differences were determined with independent samples t-tests with a 2-sided *p*-value significance cutoff of <0.05. For the adjusted analyses, univariate linear regression models were performed, with statistical significance defined as confidence intervals that excluded 0. The regression models were further adjusted for infant sex, exclusive breastfeeding, child age at sample collection, sample storage time, maternal age, smoking, maternal race and ethnicity, maternal BMI, and maternal underlying disorders as described above. Cases with missing data on covariates were excluded from the regression models.

As a data reduction technique, we also performed a principal components analysis (PCA) of the molar concentrations of individual HMOs stratified by secretor status. The optimal number of components was based on a visual determination of the elbow of the Spree curves (Supplementary Fig. S3). Multivariable linear models were repeated with each principal component, all stratified by secretor status and adjusted for the aforementioned covariates.

In the post-hoc analysis on DSLNT, the absolute concentrations of DSLNT (mmol/L) were compared between exposure groups. As the differences in DSLNT concentrations between exposure groups were similar among secretors and non-secretors, and due to small sample sizes of the new exposure groups, this post-hoc analysis was performed without stratification on secretor status. For these analyses, the same regression models were used as in the main analyses, as described above. Due to the very small number of early milk samples in the cohort, the comparison of levels of DSLNT between early and later samples was performed in the entire cohort, without studying the exposure groups separately, and also due to the imbalance of the group sizes, Mann-Whitney U test was used for this comparison. Statistical analyses were performed with SPSS Statistics for Windows, Version 29.0 (IBM Corp., Armonk, NY, Released 2022).

### Ethics

This study was approved by the University of California, San Diego Institutional Review Board (Project #130658) on December 6th, 2023. All procedures followed were in accordance with the ethical standards of the responsible committee on human experimentation (institutional and national) and with the Helsinki Declaration of 1975, as revised in 2000.

## RESULTS

### Background characteristics

Out of the 2908 participants enrolled in the HMB between 2014 and 2022, human milk oligosaccharides had previously been analyzed in 927 samples from 585 participants (Fig. 1). Out of these, 41 samples were excluded due to missing data on exposures, and 342 due to sequential samples from the same participant. In the remaining 544 samples, exposure to the included medications was assessed. Out of these, 14 (2.6%) were treated with both antidepressants and anti-inflammatory drugs and were therefore excluded from the primary analysis (Fig. 1). Therefore, the final cohort consisted of 530 participants, 136 (25.7%) of whom were treated with antidepressants; 111 (81.6%) with SSRIs, 11 (8.1%) with SNRIs, 2 (1.5%) with TCAs, and 12 (8.8%) a combination of these antidepressants. Of the 530 participants in the sample, 92 (17.4%) were treated with anti-inflammatory drugs; 50 (54.3%) with MABs, 5 (5.4%) with systemic steroids, 6 (6.5%) with traditional DMARDs, and 31 (33.7%) with a combination of these. The remaining 302 (57.0%) participants were not treated with drugs in any of these classes.

Of all the participants, 516 (97.4%) resided in the United States and 14 (2.6%) in Canada. Participants in both medication groups were more likely to be non-Hispanic White than the participants treated with neither (Table 1). Co-medications with other prescription medications other than the studied drugs were most common in the antidepressants group, 23 (16.9%), compared to 29 (9.6%) amongst the not-treated (*p* = 0.046). Amongst all infants, the mean infant age at sample collection was 0.6 years (7.2 months) and 211 (39.8%) were exclusively breastfed at the time of sample collection, but the infants of participants treated with anti-inflammatory drugs were significantly younger than the infants of unmedicated participants, and more likely to be exclusively breastfed (Table 1). Infant age at the time of sample collection did not differ between the antidepressant treated and untreated groups, but participants treated with antidepressants were more likely to give birth prematurely and less likely to breastfeed exclusively than the untreated participants.

All samples were collected with electric breast pumps. Of all the samples, 400 (88%) were full expressions of human milk, with mean (SD) collected volume 53.1 (17.9) ml, and the mean collection time being around 11am in the morning in all groups. There was no significant difference between the groups in these sample collection characteristics (Table 1). The mean (SD) sample storage time was 2.3 (1.6) years, with samples from both exposed groups stored significantly longer than unexposed samples (Table 1).

### Total HMO concentrations

Median (IQR) total concentrations of HMOs were 15.47 (4.00) mmol/L in the 390 milk samples from secretors and 9.29 (1.61) mmol/L in the 140 samples from non-secretors. In secretors, the total HMO concentrations were lower in milk samples from participants treated with antidepressants or anti-inflammatory drugs than in samples from participants treated with neither (p < 0.001 for both), but in non-secretors, the total concentrations did not differ significantly by medication use (Table 2). Median (IQR) total concentrations of HMO-bound sialic acid were 2.26 (1.93) mmol/L in secretors and 2.32 (1.95) mmol/L in non-secretors, and of HMO-bound fucose 14.05 (3.54) mmol/L in secretors and 7.20 (3.05) mmol/L in non-secretors. In both secretors and non-secretors, the total concentrations of HMO-bound sialic acid were lower in milk samples from both medication groups than from unmedicated participants (p < 0.001 for all), but the difference between groups was only statistically significant for antidepressants, among both secretors and non-secretors, after adjustments for relevant covariates (Table 2). In secretors, median total concentrations of HMO-bound fucose were lower in samples from both medication groups than from unmedicated participants (p < 0.001 for both), whereas in non-secretors, they were higher in samples from both medication groups than from untreated participants (p < 0.001 for both), but after adjustment for clinically relevant covariates, the difference between groups was only statistically significant for antidepressants and only among secretors.

### Composition of the HMOs

The crude median (IQR) relative abundance of the total of fucosylated HMOs was higher in samples from participants with than without these chronic medications in both secretors and non-secretors, 85.9 (9.7) and 82.1 (9.3) percent in secretors treated with antidepressants and anti-inflammatory drugs respectively, vs 75.7 (8.1) percent in untreated secretor positive participants (*p* < 0.001 for both) (Tables 3), and 78.9 (23.7) and 76.0 (14.5) vs 54.7 (17.6) percent in non-secretors (p < 0.001 for both) (Table 4), but after adjustment for clinically relevant covariates, these differences were only statistically significant for antidepressants. The relative abundance of the total of sialylated HMOs was lower in milk samples from both groups of medicated than from non-medicated participants, in both secretors and non-secretors (*p* < 0.001 for all), but these differences were no longer statistically significant after adjustment for clinically relevant covariates (Tables 3, 4). There was also a trend towards lower non-fucosylated, non-sialylated oligosaccharides in samples from both secretors and non-secretors, but these differences were no longer significant after adjustments (Tables 3, 4). The relative abundances (Tables 3, 4 and Supplementary Figs. S1 and S2) and absolute molar concentrations (Supplementary Tables S1 and S2) of several of the individual HMOs also varied between milk samples from medicated and unmedicated participants.

### Principal component analysis

In secretors, the HMOs were divided into three principal components (PC), explaining 55.8% of the variance, and in non-secretors in five, explaining 72.8% of the variance (Supplementary Table S3). In secretors, the levels of PC 1 (primarily LNFP II, LNFP III, DFLNT, and LNnT, loading scores between 0.7–0.8) were significantly lower in samples from participants treated with antidepressants than from untreated participants, beta estimate (95% CI) −0.7 (−1.0, −0.5). In non-secretors, the levels of PC1 (with loading scores for LNnT, LNFP III and DSLNT between 0.4–0.8 and for FLNH, 3FL and DFLNH between −0.6 - −0.8) and PC4 (mainly LNFP II, FDSLNH, DFLNT and LNH with loading scores between 0.6–0.8) were lower for milk samples from participants treated with antidepressants than from unmedicated participants, beta estimates (95% CI) −0.9 (−1.4, −0.5) and −0.9 (−1.5, −0.3) (Supplementary Tables S4 and S5). The correlation coefficients for the HMOs for each PC were in line with their loading scores (Supplementary Tables S5 and S6). The eigenvalues for the individual PCs are presented in Supplementary Table S3 and Supplementary Fig. S3, and their correlation matrix in Supplementary Table S7.

### Disialyllacto-N-tetraose (DSLNT)

The levels of DSLNT were significantly lower in samples from participants treated with antidepressants, participants with anti-inflammatory drugs and the 14 participants treated with both antidepressants and anti-inflammatory drugs, with medians (IQR) of 0.09 (0.09) mmol/L, 0.09 (0.13) mmol/L and 0.09 (0.02) mmol/L for these groups, than in samples from untreated participants with a median (IQR) DSLNT level of 0.36 (0.36) mmol/L (Supplementary Tables S1 and S2), but the adjusted beta coefficients (95% CI) compared to untreated were only significant for antidepressants, −0.09 (−0.15, −0.03) and not for anti-inflammatory drugs, 0.05 (−0.03, 0.14), or both medications, 0.07 (−0.09, 0.23). The differences were similar in secretors and non-secretors, and the levels of DSLNT in the 97 samples from participants with untreated inflammatory and/or mood disorders were similar to the levels in samples from healthy untreated participants, median (IQR) 0.31 (0.13) (Tables 3, 4, Fig. 2, Supplementary Tables S1, S2).

Of all the 530 milk samples, 46 (7.9%) were from participants who delivered preterm infants, and 11 (2.1%) of these were collected in the neonatal period, defined as before 44 weeks of gestation, i.e., within four weeks from the estimated due date, and defined as early samples. Out of these early samples, one was from a mother treated with antidepressants and one from a mother treated with anti-inflammatories, whereas the remaining nine samples were from unexposed mothers. The median (IQR) DSLNT of these 11 early samples was 0.39 (0.28) mmol/L, compared to 0.16 (0.33) mmol/L in the remaining 519 late samples expressed after 44 weeks of gestation (*p* = 0.022).

## DISCUSSION

This study found that maternal treatments with antidepressants and anti-inflammatory drugs were associated with significantly lower total concentrations of HMOs in human milk among secretors, but not among non-secretors. In both secretors and non-secretors, maternal medications were associated with increased relative abundances of several fucosylated oligosaccharides, which was statistically significant after adjustment only among antidepressant exposed. There was also a non-significant shift in HMO composition towards decreased relative abundance of sialylated and non-fucosylated, non-sialylated oligosaccharides. These associations were also confirmed by a PCA, and are in line with a recent study that showed negative correlations between maternal stress and depression and the levels of several HMOs, where the authors speculated that their results may be confounded by medication exposure.^32^

Considering that low DSLNT levels in human milk are predictive of NEC in preterm infants, the lower levels of DSLNT in samples from participants treated with chronic medications may be concerning. All mean and median concentrations of DSLNT in samples from participants with medications were below the suggested protective threshold of NEC of 0.24 mmol/L.^14,15^ However, the adjusted difference between groups was only significant for antidepressants and not anti-inflammatory drugs. Furthermore, HMO levels are suggested to be higher in general in preterm than in term milk.^7,21^ In our post-hoc analysis, we had only 11 samples that were collected in the neonatal period from mothers who delivered preterm infants, only two of which were exposed to the study medications. Thus, the association between maternal medications and DSLNT levels in human milk needs to be further studied specifically in preterm milk.

In this study, several fucosylated HMOs were found in higher proportions in human milk from participants treated with chronic medications than untreated participants, among both secretors and non-secretors. After adjustment, this difference was statistically significant for antidepressant users only. Fucosylated HMOs are known to be particularly important for the gut microbiome, and 3FL, the fucosylated HMO that varied most between groups, is also known to protect from binding of *Pseudomonas aeruginosa* to the respiratory tract.^8,29^ These differences may therefore have positive implications for the immunoprotection of the breastfed infant, but need to be weighed against any potential implications of lower proportions of other HMOs and the overall change in the complex HMO composition.

There remains a substantial knowledge gap in our understanding of the overall variation in HMO composition within an individual’s milk supply and any potential clinical effects on the breastfed infant. Similarly, the determinants and potential modifiers of HMO composition are not well understood. In this study, we chose to analyze specific HMOs separately and then to group them based on their fucosylation and sialylation. However, whether and how specific HMOs or some combination of HMOs directly affect the breastfed infant across the life course, including on their immune systems and microbiota, requires further study.^1,2,13,38^

### Strengths and limitations

The major strengths of this study were the quantitative methods of the HMO analysis, the large sample of participants treated and not treated with chronic medications, the regression analyses used to adjust for multiple fixed, modifiable, and environmental factors, and the PCA analysis.

Even though we had a large number of human milk samples from 530 participants, the interpretation of the results for non-secretors is likely to be underpowered due to the limited number of non-secretors. As only 5–40% of lactating individuals are non-secretors, this was expected and is a common dilemma. Similarly, we had a smaller number of anti-inflammatory-treated than antidepressant-treated mothers with more heterogeneity in the specific anti-inflammatory medications used. This could have resulted in insufficient power for some comparisons. Interpretation of differences in HMO concentrations for individual medications, and the interpretation of the effects on DSLNT in preterm milk, were also limited by the sample size. Another limitation was the different mean storage times of the exposed and unexposed samples. While storage time was included as a covariate in the adjusted analyses, the direct effect of storage time on the HMO composition of human milk is yet largely unstudied. This adds uncertainty into the interpretation of the crude and adjusted results. Furthermore, we could not adequately account for any potential effects that underlying disease severity or diet may have on the HMO composition. Finally, a limitation to the PCA is that the levels of HMOs are likely to be correlated with each other.^32^ To account for this, a direct oblimin method was used, allowing for interaction between the components.

## CONCLUSIONS

This study found that treatment with antidepressants and anti-inflammatory drugs was associated with lower total concentrations of HMOs in human milk from secretors, higher proportions of several fucosylated HMOs, particularly among antidepressant users, and a trend toward lower non-fucosylated, non-sialylated, and sialylated HMOs in both secretors and non-secretors. Human milk from participants treated with antidepressants also had lower concentrations of DSLNT, a sialylated oligosaccharide potentially protective of NEC. Larger studies are needed to confirm these findings, and the clinical relevance for the breastfed infant of variations of this type and magnitude in HMO composition is yet to be determined. Hence, these findings do not discourage from use of antidepressants and anti-inflammatories during lactation, if medically indicated.

## Supplementary Material

Supplementary Material

ADDITIONAL INFORMATION

**Supplementary information** The online version contains supplementary material available at https://doi.org/10.1038/s41390-025-04650-5.

## Figures and Tables

**Table 1.** Background characteristics of participants

	Antidepressants *n* = 136	Anti-inflammatory drugs *n* = 92	Neither medication *n* = 302
Maternal characteristics			
Age, years, mean, (SD)	33.4 (4.3)	32.7 (3.9)	33.1 (4.4)
BMI, kg/m^2^ mean, (SD)	28.1 (7.0)*	24.9 (4.3)	26.0 (8.1)
Race & ethnicity, *n* (%)			
Hispanic	10 (7.4)*	6 (6.5)**	38 (12.6)
Non-Hispanic Black	3 (2.2)*	0 (0.0)**	5 (1.5)
Non-Hispanic White	121 (89.0)*	83 (90.2)**	232 (76.8)
Other^a^	2 (1.5)*	3 (3.3)**	27 (8.9)
Tobacco use, *n* (%)	5 (3.7)*	0 (0.0)**	8 (2.6)
Mood disorder composite, *n* (%)			
Psychiatric diagnosis and EPDS ≥ 10p	36 (26.5)*	3 (3.3)**	12 (4.0)
EPDS ≥ 10p or a mood disorder	87 (64.0)*	14 (15.2)**	61 (20.2)
No psychiatric diagnosis, EPDS < 10p	13 (9.6)*	75 (81.5)**	229 (75.8)
Inflammatory disorders, *n* (%)	16 (11.8)	86 (93.5)**	33 (10.9)
Co-medications,^b^ *n* (%)	23 (16.9)*	9 (9.8)	29 (9.6)
Infant characteristics			
Age, years (mean, SD)	0.7 (0.5)	0.4 (0.4)**	0.7 (0.7)
Sex male, *n* (%)	77 (56.6)	50 (54.3)	165 (54.8)
Preterm, *n* (%)	18 (13.2)*	7 (7.6)	21 (7.0)
Feeding, *n* (%)			
Exclusive human milk	39 (28.7)*	52 (56.5)**	120 (40.1)
Human milk and infant formula	16 (11.8)*	16 (17.4)**	17 (5.7)
Human milk and solid foods	69 (50.7)*	19 (20.7)**	150 (50.2)
Human milk, formula and solids	12 (8.8)*	5 (5.4)**	12 (4.0)
Sample collection data			
Full expression, *n* (%)	94 (89.5)	61 (89.7)	245 (87.2)
Collection time, hours, mean (SD)	11.7 (3.8)	11.8 (4.5)	11.3 (3.8)
Volume, ml (mean, SD)	50.9 (20.1)	52.1 (17.3)	54.4 (17.0)
Storage time, years, mean (SD)	3.1 (1.8)*	3.9 (1.7)**	1.5 (0.7)

* *p*-value significant (*p* < 0.05) between samples from antidepressant exposed and unexposed participants.

** *p* value significant (*p* < 0.05) between samples from anti-inflammatory exposed and unexposed participants.

Missing data: maternal age (*n* = 2), infant sex (*n* = 1), BMI (*n* = 3), infant feeding (*n* = 3), EPDS (*n* = 71), full expression (*n* = 76), sample collection time (*n* = 55).

a other including asian, american indian, native hawaiian/pacific islander.

b with other prescription medications than antidepressants and anti-inflammatory drugs.

*BMI* Body Mass Index, *SD* Standard Deviation, *EPDS* Edinburgh Postnatal Depression Scale.

**Table 2.** Total Concentrations of Human Milk Oligosaccharides, Sialic Acid and Fucose in Human Milk from Participants Treated with Antidepressants, Anti-inflammatory Drugs or Neither

	Antidepressants *n* = S 99 /NS 37		Anti-inflammatory drugs *n* = S 58 / NS 34		Neither *n* = S 233 / NS 69		Beta coefficient with 95% CI, Adjusted model	
	Mean (SD)	Median (IQR)	Mean (SD)	Median (IQR)	Mean (SD)	Median (IQR)	Antidepressants vs Neither^a^	Anti-inflammatory D›rugs vs Neither^b^
Secretors								
Total HMOs, mmol/L	12.95* (3.61)	12.43 (5.32)	12.80** (3.15)	12.71 (4.94)	16.06 (2.06)	16.10 (2.25)	−1.62 (−2.44, −0.79)	−1.12 (2.16, −0.08)
HMO-bound Sia, mmol/L	1.48* (1.06)	1.00 (1.14)	1.71** (0.95)	1.36 (1.17)	2.77 (0.95)	2.84 (1.18)	−0.39(−0.69, −0.09)	−0.03 (−0.46, 0.39)
HMO-bound Fuc, mmol/L	12.77* (4.04)	12.27 (4.20)	11.97** (2.82)	12.25 (4.92)	14.82 (2.27)	14.74 (2.47)	−1.20 (−2.14, −0.27)	−0.94 (−2.05, 0.18)
Total HMOs, mg/L	8.37* (2.56)	7.85 (4.82)	8.57** (2.41)	8.41 (4.82)	11.11 (1.15)	11.40 (0.70)	−1.39 (−1.90, −0.89)	−0.70 (−1.31, −0.10)
Non-Secretors								
Total HMOs, mmol/L	10.48 (3.34)	9.65 (3.15)	10.05 (2.43)	9.58 (3.05)	9.42 (0.87)	9.23 (0.62)	0.28 (−0.97, 1.53)	0.21 (−1.10, 1.53)
HMO-bound Sia, mmol/L	1.69* (1.03)	1.29 (1.18)	1.86** (0.97)	1.75 (1.44)	2.96 (0.87)	3.04 (1.05)	−0.61 (−1.11, −0.11)	0.02 (−0.61, 0.66)
HMO-bound Fuc, mmol/L	8.61* (3.39)	7.90 (4.66)	8.39** (2.58)	8.19 (2.95)	6.29 (2.00)	6.70 (2.28)	0.32 (−1.07, 1.70)	0.62 (−1.15, 2.39)
Total HMOs, mg/L	7.30 (2.45)	6.91 (2.34)	7.18** (1.78)	7.51 (2.23)	8.13 (0.36)	8.09 (0.27)	−0.66 (−1.54, 0.22)	0.03 (−0.81, 0.87)

* *p*-value significant (*p* < 0.05) between samples from antidepressant exposed and unexposed participants.

** *p*-value significant (*p* < 0.05) between samples from anti-inflammatory-exposed and unexposed participants.

Bold denotes confidence intervals not crossing 0.

a model adjusted for infant sex, exclusive breastfeeding, child collection age, maternal age, smoking, race and ethnicity, maternal body mass index and a composite variable for maternal mood, consisting of current mood disorders and depressive symptoms measured with the Edinburgh Postnatal Depression Scale.

b model adjusted for infant sex, exclusive breastfeeding, child collection age, maternal age, smoking, race and ethnicity, maternal body mass index and maternal diagnosis of inflammatory disorders.

*S* Secretors, *NS* Non-Secretors, *CI* Confidence Interval, *IQR* Interquartile Range, *HMO* Human Milk Oligosaccharide, *Sia* Sialic Acid, *Fuc* Fucose.

**Table 3.** Relative Abundances of Individual Human Milk Oligosaccharides (HMOs) in Human Milk from Secretors Treated with Antidepressants, Anti-inflammatory drugs or Neither, Measured in Percentage of Total HMO Concentration

HMO	Antidepressants, *n* = 99		Anti-inflammatory drugs, *n* = 58		Neither medication, *n* = 233		Beta coefficient with 95% CI, Adjusted model	
	Mean (SD)	Median (IQR)	Mean (SD)	Median (IQR)	Mean (SD)	Median (IQR)	Anti-depressants vs Neither^a^	AIDs vs Neither^b^
Non-fucosylated, Non-sialylated Oligosaccharides								
LNnT	1.57* (1.83)	0.88 (1.28)	1.84** (1.64)	1.40 (1.42)	3.38 (2.28)	3.08 (3.16)	−1.1 (−1.7, −0.4)	−0.7 (−1.7, 0.2)
LNT	6.14 (3.97)	5.40 (4.67)	7.56 (3.27)	6.88 (3.85)	6.87 (4.23)	6.07 (5.12)	−0.3 (−1.7, 1.0)	0.3 (−1.7, 2.2)
LNH	0.43 (0.31)	0.33 (0.32)	0.48 (0.27)	0.44 (0.41)	0.48 (0.44)	0.42 (0.31)	−0.0 (−0.2, 0.1)	−0.3 (−0.5, −0.1)
Total	8.14* (5.07)	6.97 (6.23)	9.88 (4.12)	9.46 (5.64)	10.73 (5.90)	9.81 (7.2)	−1.4 (−3.3, 0.4)	−0.8 (−3.4, 1.9)
Fucosyl-oligosaccharides								
2′FL	37.28 (11.96)	34.35 (16.77)	37.26 (11.79)	36.02 (14.36)	40.07 (13.26)	40.48 (16.95)	−2.3 (−6.6, 2.0)	−3.0 (−9.3, 3.3)
3FL	20.68* (13.61)	21.42 (22.87)	16.58** (12.26)	17.03 (21.57)	4.55 (7.21)	2.84 (1.69)	8.5 (5.7, 11.4)	3.6 (−0.1, 7.4)
DFLac	6.59* (5.28)	5.67 (3.70)	4.81 (2.5)	4.36 (3.45)	4.39 (3.12)	3.49 (3.53)	1.5 (0.3, 2.7)	0.8 (−0.6, 2.2)
LNFP I	4.3* (3.2)	3.39 (2.93)	5.83 (4.37)	4.49 (4.44)	5.75 (3.58)	4.78 (4.46)	−0.7 (−1.9, 0.4)	0.5 (−1.3, 2.3)
LNFP II	6.73* (3.04)	6.49 (4.20)	7.55** (3.23)	7.27 (4.01)	10.19 (5.48)	9.59 (6.76)	−3.2 (−4.8, −1.6)	−1.5 (−4.0, 1.0)
LNFP III	0.2* (0.19)	0.12 (0.14)	0.20** (0.16)	0.14 (0.14)	0.51 (0.29)	0.45 (0.33)	−0.2 (−0.3, −0.1)	−0.2 (−0.4, −0.1)
DFLNT	6.74* (3.32)	6.34 (2.64)	6.90** (3.96)	6.68 (3.71)	9.87 (4.34)	10.23 (4.96)	−1.4 (−2.8, −0.1)	0.5 (−1.5, 2.6)
FLNH	0.74* (0.72)	0.56 (0.64)	0.93** (0.68)	0.83 (0.80)	0.40 (0.43)	0.24 (0.37)	0.3 (0.2, 0.5)	0.0 (−0.2, 0.3)
DFLNH	0.47* (0.36)	0.43 (0.30)	0.60** (0.47)	0.54 (0.46)	0.33 (0.38)	0.19 (0.31)	0.2 (0.0*, 0.3)	0.0 (−0.2, 0.2)
Total	83.75* (8.03)	85.89 (9.68)	80.65** (6.83)	82.05 (9.26)	76.04 (7.38)	75.7 (8.14)	2.7 (0.4, 5.1)	0.8 (−2.6, 4.2)
Sialyl-oligosaccharides								
3′SL	2.82* (3.31)	1.88 (1.56)	2.64** (2.41)	1.76 (1.77)	6.46 (4.51)	5.78 (4.83)	−1.5 (−2.7, −0.2)	−0.7 (−2.6, 1.1)
6′SL	1.58 (1.48)	1.22 (0.97)	2.19** (1.42)	1.80 (2.03)	1.49 (1.46)	1.09 (1.10)	0.7 (0.2, 1.1)	0.3 (−0.3, 0.9)
LSTb	0.62 (0.26)	0.59 (0.31)	0.76 (0.37)	0.72 (0.36)	0.67 (0.35)	0.60 (0.41)	−0.0 (−0.1, 0.1)	0.1 (−0.1, 03)
LSTc	0.41 (0.39)	0.28 (0.32)	0.63 (0.45)	0.42 (0.57)	0.43 (0.46)	0.26 (0.34)	0.1 (−0.0, 0.2)	0.1 (−0.1, 03)
DSLNT	1.07* (0.87)	0.79 (0.59)	1.32** (1.18)	0.92 (0.95)	2.19 (1.62)	1.95 (2.14)	−0.5 (−0.9, −0.0*)	0.4 (−0.3, 1.1)
DSLNH	0.43 (0.55)	0.27 (0.34)	0.61** (0.45)	0.46 (0.60)	0.38 (0.43)	0.23 (0.42)	0.2 (0.1, 0.3)	0.1 (−0.0, 0.3)
Total	6.92* (4.37)	5.36 (3.56)	8.15** (3.69)	7.2 (4.58)	11.62 (4.82)	11.48 (5.85)	−1.0 (−2.5, 0.5)	0.4 (−1.8, 2.5)
Fucosyl- and Sialyl-oligosaccharides								
FDSLNH	1.19* (0.8)	1.02 (0.79)	1.32** (0.64)	1.24 (0.76)	1.60 (1.09)	1.39 (1.11)	−0.3, −0.6, 0.0)	−0.4 (−0.9, 0.1)

* *p* value significant (*p* < 0.05) between samples from antidepressant exposed and unexposed participants.

** *p* value significant (*p* < 0.05) between samples from anti-inflammatory exposed and unexposed participants.

a model adjusted for infant sex, exclusive breastfeeding, child collection age, maternal age, smoking, race and ethnicity, maternal body mass index and a composite variable for maternal mood, consisting of current mood disorders and depressive symptoms measured with EPDS. Bold denotes confidence intervals not crossing 0.

b model adjusted for infant sex, exclusive breastfeeding, child collection age, maternal age, smoking, race and ethnicity, maternal body mass index and maternal diagnosis of inflammatory disorders. Bold denotes confidence intervals not crossing 0.

*HMO* Human Milk Oligosaccharide, *SD* Standard Deviation, *IQR* Interquartile range, *AID* Anti-inflammatory drugs, *CI* Confidence Interval, *LNnT* lacto-N-neotetraose, *LNT* lacto-N-tetraose, *LNH* lacto-N-hexaose, *2′FL2′*-fucosyllactose, *3FL* 3-fucosyllactose, *DFLac* difucosyllactose, *LNFP I* lacto-N-fucopentaose I, *LNFP II* lacto-N-fucopentaose II, *LNFP III* lacto-N-fucopentaose III, *DFLNT* difucosyl-lacto-N-tetraose, *FLNH* fucosyllacto-N-hexaose, *DFLNH* difucosyl-lacto-N-hexaose, *3′SL* = 3′-sialyllactose, *6′SL* = 6′-sialyllactose, *LSTb* sialyl-lacto-N-tetraose b, *LSTc* sialyl-lacto-N-tetraose c, *DSLNT* disialyllacto-N-tetraose, *DSLNH* disialyl-lacto-Nhexaose, *FDSLNH* fucosyl-disialyl-lacto-N-hexaose.

**Table 4.** Relative Abundances of Individual Human Milk Oligosaccharides (HMOs) in Human Milk from Non-Secretors Treated with Antidepressants, Anti-inflammatory drugs or Neither, Measured in Percentage of Total HMO Concentration

HMO	Antidepressants, *n* = 37		Anti-inflammatory Drugs, *n* = 34		Neither medication, *n* = 69		Beta coefficient with 95% CI, Adjusted model	
	Mean (SD)	Median (IQR)	Mean (SD)	Median (IQR)	Mean (SD)	Median (IQR)	Antidepressants vs Neither^a^	AIDs vs Neither^b^
Non-fucosylated, non-sialylated oligosaccharides								
LNnT	1.98* (4.38)	0.62 (0.89)	1.14** (1.83)	0.56 (0.44)	7.28 (5.03)	6.59 (6.09)	−3.5 (−6.3, −0.7)	−3.1 (−6.5, 0.2)
LNT	12.76* (10.77)	9.88 (9.35)	11.83** (8.05)	9.78 (9.1)	17.78 (9.14)	17.02 (12.84)	1.8 (−3.4, 7.0)	−1.2 (−7.9, 5.6)
LNH	0.59* (0.53)	0.38 (0.47)	0.58** (0.52)	0.47 (0.33)	1.00 (0.65)	0.90 (0.63)	−0.3 (−0.7, 0.0)	0.3 (−0.1, 0.7)
Total	15.32* (12.73)	10.87 (13.72)	13.56** (9.09)	12.36 (10.16)	26.07 (12.62)	23.53 (15.12)	−2.0 (−8.7, 4.6)	−4.0 (−12.8, 4.8)
Fucosyl-oligosaccharides								
2′FL	1.11 (2.01)	0.49 (0.62)	0.75** (0.87)	0.53 (0.63)	0.43 (0.55)	0.22 (0.52)	1.0 (0.3, 1.8)	0.2 (−0.4, 0.8)
3FL	46.51* (25.67)	54.41 (37.75)	46.27** (21.72)	50.54 (22.73)	5.44 (13.25)	2.57 (2.5)	21.5 (13.1, 29.9)	9.1 (−0.1, 19.1)
DFLac	0.34* (0.25)	0.24 (0.28)	0.40 (0.51)	0.26 (0.29)	0.54 (0.5)	0.43 (0.37)	−0.0 (−0.3, 0.2)	0.0 (−0.4, 0.5)
LNFP I	1.72 (1.12)	1.51 (0.89)	1.59** (0.52)	1.49 (0.93)	1.98 (1.03)	1.86 (1.37)	0.3 (−0.3, 0.9)	0.1 (−0.6, 0.8)
LNFP II	16.87* (9.28)	13.85 (6.72)	17.36** (7.62)	16.44 (6.18)	32.85 (9.41)	34.73 (8.91)	−12.5 (−17.8, −7.1)	−4.6 (−11.2, 2.1)
LNFP III	0.28* (0.37)	0.13 (0.22)	0.28** (0.40)	0.14 (0.07)	1.30 (0.66)	1.15 (0.81)	−0.8 (−1.2, −0.5)	−0.4 (−0.9, 0.0)
DFLNT	4.00* (3.85)	2.80 (2.85)	3.83** (4.15)	2.67 (1.69)	8.80 (5.44)	8.49 (8.25)	−4.3 (−7.1, −1.6)	−1.0 (−4.5, 2.6)
FLNH	1.27* (1.36)	0.68 (1.26)	1.94** (2.25)	1.15 (1.95)	0.50 (0.58)	0.30 (0.39)	1.2 (0.7, 1.7)	1.2 (0.1, 2.3)
DFLNH	0.86* (0.5)	0.81 (0.88)	1.03** (0.51)	1.06 (0.75)	0.30 (0.32)	0.20 (0.18)	0.4 (0.2, 0.6)	0.2 (−1.3, 0.5)
Total	72.95* (17.01)	78.86 (23.65)	73.45** (13.14)	76.01 (14.45)	52.12 (15.31)	54.72 (17.55)	6.8 (−1.1, 14.7)	4.9 (−5.6, 15.3)
Sialyl-oligosaccharides								
3′SL	3.04* (2.05)	2.31 (1.72)	3.07** (2.72)	2.03 (1.39)	5.72 (3.93)	4.26 (5.16)	−1.1 (−2.8, 0.6)	0.3 (−2.1, 2.6)
6′SL	2.30* (2.01)	2.01 (1.99)	2.51** (1.64)	2.45 (1.81)	3.62 (3.67)	2.13 (3.79)	−0.4 (−2.0, 1.4)	−1.4 (−3.8, 0.9)
LSTb	1.21* (1.04)	0.92 (0.7)	1.06** (0.48)	0.96 (0.65)	1.78 (0.64)	1.78 (0.88)	−0.1 (−0.5, 0.4)	−0.1 (−0.6, 0.3)
LSTc	0.29* (0.24)	0.21 (0.22)	0.36 (0.25)	0.28 (0.42)	0.51 (0.55)	0.30 (0.45)	−0.1 (−0.3, 0.2)	−0.2 (−0.5, 0.2)
DSL NT	1.4* (1.64)	0.88 (0.50)	1.60** (1.77)	0.89 (0.87)	4.21 (2.92)	4.31 (4.37)	−1.2 (−2.7, 0.2)	−0.0 (−2.0, 1.9)
DSLNH	0.51* (0.46)	0.33 (0.41)	0.76 (0.88)	0.60 (0.86)	0.86 (0.83)	0.53 (0.95)	−0.2 (−0.5, 0.1)	0.1 (−0.5, 0.6)
Total	8.75* (4.54)	7.66 (6.12)	9.36** (5.44)	7.62 (4.43)	16.70 (7.00)	17.25 (10.57)	−3.1 (−6.5, 0.4)	−1.4 (−6.4, 3.5)
Fucosyl- and Sialyl-oligosaccharides								
FDSLNH	2.97* (2.48)	2.45 (2.35)	3.63** (2.16)	3.45 (2.5)	5.11 (2.69)	5.14 (3.64)	−1.7 (−3.1, −0.2)	0.5 (−1.3, 2.4)

* *p* value significant (*p* < 0.05) between samples from antidepressant exposed and unexposed participants.

** *p* value significant (*p* < 0.05) between samples from anti-inflammatory exposed and unexposed participants.

a model adjusted for infant sex, exclusive breastfeeding, child collection age, maternal age, smoking, race and ethnicity, maternal body mass index and a composite variable for maternal mood, consisting of current mood disorders and depressive symptoms measured with EPDS. Bold denotes confidence intervals not crossing 0.

b model adjusted for infant sex, exclusive breastfeeding, child collection age, maternal age, smoking, race and ethnicity, maternal body mass index and maternal diagnosis of inflammatory disorders. Bold denotes confidence intervals not crossing 0.

*HMO* Human Milk Oligosaccharide, *SD* Standard Deviation, *IQR* Interquartile range, *AID* Anti-inflammatory drugs, *CI* Confidence Interval, *LNnT* lacto-N-neotetraose, *LNT* lacto-N-tetraose, *LNH* lacto-N-hexaose, *2′FL* 2′-fucosyllactose, *3FL* 3-fucosyllactose, *DFLac* difucosyllactose, *LNFP I* lacto-N-fucopentaose I, *LNFP II* lacto-N-fucopentaose II, *LNFP III* lacto-N-fucopentaose III, *DFLNT* difucosyl-lacto-N-tetraose, *FLNH* fucosyl-lacto-N-hexaose, *DFLNH* difucosyl-lacto-N-hexaose, *3′SL3′*-sialyllactose, *6′SL6′*-sialyllactose, *LSTb* sialyl-lacto-N-tetraose b, *LSTc* sialyl-lacto-N-tetraose c, *DSLNT* disialyllacto-N-tetraose, *DSLNH* disialyl-lacto-N-hexaose, *FDSLNH* fucosyl-disialyl-lacto-N-hexaose.

## Data Availability

The data that supports the findings of this study can be requested from the UC San Diego Human Milk Biorepository -Principal Investigator C Chambers.

## IMPACT:

- In human milk, maternal treatment with antidepressants and anti-inflammatories was associated with higher levels of several fucosylated oligosaccharides, particularly among antidepressant users, a trend toward lower sialylated and non-fucosylated, non-sialylated oligosaccharide levels, and lower total human milk oligosaccharide concentrations among secretors.
- It is unknown whether these associations are causal, and the overall clinical implications of these findings for the breastfed infant are not clear. Potential effects of maternal mood and inflammatory disorders also need to be clarified.
- These findings should not discourage the use of anti-inflammatory or antidepressant medication during lactation when the use is medically indicated.
