# Supplementary material for: Varied human milk oligosaccharides in human milk from mothers treated with antidepressants and anti-inflammatories
Source: Pediatr Res. Author manuscript; Available in PMC 2026 Mar 23. (PMC13007041; doi:10.1038/s41390-025-04650-5)
Supplement: Supplementary Material [file NIHMS2144404-supplement-Supplementary_Material.pdf]

# Supplementary Material for:

# Varied human milk oligosaccharides in human milk from mothers treated with antidepressants and anti-inflammatories

Essi Whaites Heinonen,[1,2] Gretchen Bandoli,[1,3] Bianca Robertson,[1,4] Chloe Yonemitsu,[1,4] Lars Bode,[1,3,4] Hannah Riedy,[1,5] Kerri Bertrand,[1] Christina Chambers[1,3]

[1] Department of Pediatrics, University of California San Diego, La Jolla, California
[2] Division for Pediatrics, Department of Clinical Science, Intervention and Technology, Karolinska Institutet, Stockholm, Sweden
[3] Human Milk Institute (HMI), University of California San Diego, La Jolla, California
[4] Larsson-Rosenquist Foundation Mother-Milk-Infant Center of Research Excellence (LRF MOMI CORE), University of California San Diego, La Jolla, California
[5] Department of Pediatrics, School of Medicine, Duke University, Durham, North Carolina

**Supplementary Table S1. Mean and median molar concentrations of the individual HMOs in secretors**

| HMO, mmol/L | Antidepressants, n=99 Mean (SD) | Antidepressants, n=99 Median (IQR) | Anti-inflammatory drugs, n=58 Mean (SD) | Anti-inflammatory drugs, n=58 Median (IQR) | Neither medication, n=233 Mean (SD) | Neither medication, n=233 Median (IQR) |
|---|---|---|---|---|---|---|
| **Non-fucosylated, non-sialylated oligosaccharides** | | | | | | |
| LNnT | 0.22 (0.28)* | 0.10 (0.19) | 0.26 (0.27)** | 0.18 (0.23) | 0.54 (0.35) | 0.50 (0.48) |
| LNT | 0.78 (0.52)* | 0.62 (0.59) | 0.98 (0.53) | 0.85 (0.73) | 1.07 (0.60) | 0.99 (0.74) |
| LNH | 0.06 (0.05)* | 0.04 (0.04) | 0.06 (0.04) | 0.05 (0.06) | 0.07 (0.06) | 0.07 (0.05) |
| Total | 1.06 (0.74)* | 0.79 (0.78) | 1.30 (0.71)** | 1.19 (0.89) | 1.68 (0.84) | 1.58 (1.02) |
| **Fucosyl-oligosaccharides** | | | | | | |
| 2'FL | 4.87 (2.21)* | 4.54 (2.86) | 4.84 (2.15)** | 4.42 (2.86) | 6.62 (2.81) | 6.31 (3.65) |
| 3FL | 2.48 (1.73)* | 2.25 (2.52) | 1.88 (1.36)** | 1.8 (2.41) | 0.67 (0.88) | 0.44 (0.28) |
| DFLac | 0.90 (1.09) | 0.66 (0.43) | 0.6 (0.35) | 0.55 (0.41) | 0.70 (0.50) | 0.54 (0.55) |
| LNFP I | 0.57 (0.51)* | 0.41 (0.40) | 0.81 (0.78) | 0.54 (0.65) | 0.94 (0.64) | 0.75 (0.76) |
| LNFP II | 0.87 (0.50)* | 0.73 (0.51) | 0.97 (0.52)** | 0.84 (0.61) | 1.58 (0.72) | 1.58 (0.99) |
| LNFP III | 0.03 (0.03)* | 0.01 (0.02) | 0.03 (0.03)** | 0.02 (0.03) | 0.08 (0.04) | 0.07 (0.05) |
| DFLNT | 0.90 (0.59)* | 0.73 (0.63) | 0.91 (0.65)** | 0.73 (0.73) | 1.56 (0.67) | 1.65 (0.69) |
| FLNH | 0.09 (0.09)* | 0.06 (0.07) | 0.12 (0.09)** | 0.09 (0.11) | 0.06 (0.06) | 0.04 (0.06) |
| DFLNH | 0.06 (0.06) | 0.05 (0.04) | 0.08 (0.07)** | 0.07 (0.07) | 0.05 (0.06) | 0.03 (0.05) |
| Total | 10.79 (3.03)* | 10.36 (3.17) | 10.24 (2.39)** | 10.12 (3.56) | 12.26 (2.32) | 12.09 (2.89) |
| **Sialyl-oligosaccharides** | | | | | | |
| 3'SL | 0.40 (0.59)* | 0.22 (0.22) | 0.36 (0.39)** | 0.20 (0.25) | 1.05 (0.05) | 0.90 (0.82) |
| 6'SL | 0.21 (0.24) | 0.14 (0.14) | 0.29 (0.21) | 0.23 (0.30) | 0.24 (0.02) | 0.17 (0.17) |
| LSTb | 0.08 (0.04)* | 0.07 (0.04) | 0.10 (0.05) | 0.09 (0.06) | 0.11 (0.00) | 0.10 (0.06) |
| LSTc | 0.06 (0.06) | 0.03 (0.04) | 0.08 (0.07) | 0.06 (0.10) | 0.07 (0.00) | 0.04 (0.06) |
| DSLNT | 0.15 (0.14)* | 0.09 (0.10) | 0.18 (0.19)** | 0.11 (0.16) | 0.34 (0.24) | 0.33 (0.35) |
| DSLNH | 0.06 (0.08) | 0.03 (0.04) | 0.08 (0.07)** | 0.06 (0.10) | 0.06 (0.07) | 0.04 (0.07) |
| Total | 0.95 (0.79)* | 0.65 (0.67) | 1.09 (0.66)** | 0.89 (0.87) | 1.87 (0.80) | 1.80 (0.98) |
| **Fucosyl- and Sialyl-oligosaccharides** | | | | | | |
| FDSLNH | 0.16 (0.12)* | 0.12 (0.10) | 0.17 (0.01)** | 0.15 (0.11) | 0.25 (0.14) | 0.22 (0.18) |

* p value significant (p<0.05) between samples from antidepressant exposed and unexposed participants
** p value significant (p<0.05) between samples from anti-inflammatory exposed and unexposed participants
HMO= Human Milk Oligosaccharide, SD= Standard Deviation, IQR= Interquartile range, LNnT= lacto-N-neotetraose, LNT= lacto-N-tetraose, LNH= lacto-N-hexaose, 2'FL= 2'-fucosyllactose, 3FL= 3'-fucosyllactose, DFLac= difucosyllactose, LNFP I= lacto-N-

fucopentaose I, LNFP II= lacto-N-fucopentaose II, LNFP III= lacto-N-fucopentaose III, DFLNT= difucosyl-lacto-N-tetraose, FLNH= fucosyl-lacto-N-hexaose, DFLNH= difucosyl-lacto-N-hexaose, 3'SL= 3'-sialyllactose, 6'SL= 6'-sialyllactose, LSTb= sialyl-lacto-N-tetraose b, LSTc= sialyl-lacto-N-tetraose c, DSLNT= disialyllacto-N-tetraose, DSLNH= disialyl-lacto-N-hexaose, FDSLNH= fucosyl-disialyl-lacto-N-hexaose

**Supplementary Table S2. Mean and median molar concentrations of the individual HMOs in non-secretors**

| HMO, mmol/L | Antidepressants, n=37 | | Anti-inflammatory drugs, n=34 | | Neither medication, n=69 | |
|---|---|---|---|---|---|---|
| | Mean (SD) | Median (IQR) | Mean (SD) | Median (IQR) | Mean (SD) | Median (IQR) |
| **Non-fucosylated, non-sialylated oligosaccharides** | | | | | | |
| LNnT | 0.19 (0.42)* | 0.07 (0.07) | 0.11 (0.17)** | 0.05 (0.05) | 0.69 (0.5) | 0.61 (0.58) |
| LNT | 1.27 (1.18)* | 0.99 (0.97) | 1.12 (0.63)** | 1.02 (0.93) | 1.68 (0.92) | 1.53 (1.25) |
| LNH | 0.06 (0.05)* | 0.03 (0.04) | 0.06 (0.05)** | 0.04 (0.04) | 0.09 (0.06) | 0.08 (0.06) |
| Total | 1.51 (1.36)* | 1.11 (1.25) | 1.29 (0.74)** | 1.14 (1.06) | 2.46 (1.29) | 2.14 (1.43) |
| **Fucosyl-oligosaccharides** | | | | | | |
| 2'FL | 0.09 (0.14)* | 0.06 (0.06) | 0.07 (0.07)** | 0.05 (0.07) | 0.04 (0.05) | 0.02 (0.04) |
| 3FL | 5.03 (3.23)* | 4.96 (4.83) | 4.77 (2.61)** | 4.98 (2.74) | 0.59 (1.64) | 0.23 (0.24) |
| DFLac | 0.03 (0.02)* | 0.03 (0.02) | 0.04 (0.05) | 0.02 (0.04) | 0.05 (0.06) | 0.04 (0.04) |
| LNFP I | 0.18 (0.16) | 0.16 (0.09) | 0.16 (0.06) | 0.16 (0.10) | 0.19 (0.1) | 0.18 (0.11) |
| LNFP II | 1.74 (1.02)* | 1.44 (1.57) | 1.74 (0.78)** | 1.68 (1.15) | 3.06 (0.85) | 3.25 (0.95) |
| LNFP III | 0.03 (0.03)* | 0.01 (0.01) | 0.03 (0.04)** | 0.01 (0.01) | 0.12 (0.07) | 0.11 (0.08) |
| DFLNT | 0.43 (0.39)* | 0.32 (0.43) | 0.38 (0.37)** | 0.27 (0.27) | 0.81 (0.49) | 0.79 (0.73) |
| FLNH | 0.13 (0.17)* | 0.07 (0.12) | 0.19 (0.2)** | 0.10 (0.20) | 0.05 (0.05) | 0.03 (0.04) |
| DFLNH | 0.09 (0.07)* | 0.07 (0.08) | 0.11 (0.07)** | 0.11 (0.12) | 0.03 (0.03) | 0.02 (0.02) |
| Total | 7.76 (3.22)* | 7.26 (4.66) | 7.48 (2.47)** | 7.56 (3.16) | 4.93 (1.77) | 5.08 (1.68) |
| **Sialyl-oligosaccharides** | | | | | | |
| 3'SL | 0.31 (0.22)* | 0.24 (0.23) | 0.29 (0.25)** | 0.22 (0.11) | 0.53 (0.37) | 0.39 (0.46) |
| 6'SL | 0.25 (0.31) | 0.17 (0.22) | 0.25 (0.17) | 0.24 (0.26) | 0.34 (0.36) | 0.2 (0.36) |
| LSTb | 0.13 (0.13)* | 0.09 (0.08) | 0.1 (0.05)** | 0.09 (0.08) | 0.17 (0.06) | 0.16 (0.08) |
| LSTc | 0.03 (0.03) | 0.02 (0.02) | 0.04 (0.03) | 0.03 (0.05) | 0.05 (0.05) | 0.03 (0.04) |
| DSLNT | 0.14 (0.15)* | 0.09 (0.10) | 0.15 (0.16)** | 0.09 (0.11) | 0.39 (0.27) | 0.38 (0.41) |
| DSLNH | 0.05 (0.05)* | 0.03 (0.05) | 0.08 (0.08) | 0.06 (0.09) | 0.08 (0.07) | 0.05 (0.09) |
| Total | 0.91 (0.57)* | 0.74 (0.69) | 0.92 (0.5)** | 0.82 (0.73) | 1.56 (0.66) | 1.57 (1.05) |
| **Fucosyl- and Sialyl-oligosaccharides** | | | | | | |
| FDSLNH | 0.3 (0.25)* | 0.21 (0.23) | 0.36 (0.22)** | 0.36 (0.31) | 0.47 (0.23) | 0.48 (0.31) |

* p value significant ($p<0.05$) between samples from antidepressant exposed and unexposed participants
** p value significant ($p<0.05$) between samples from anti-inflammatory exposed and unexposed participants
HMO= Human Milk Oligosaccharide, SD= Standard Deviation, IQR= Interquartile range, LNnT= lacto-N-neotetraose, LNT= lacto-N-tetraose, LNH= lacto-N-hexaose, 2'FL= 2'-fucosyllactose, 3FL= 3'-fucosyllactose, DFLac= difucosyllactose, LNFP I= lacto-N-

fucopentaose I, LNFP II= lacto-N-fucopentaose II, LNFP III= lacto-N-fucopentaose III, DFLNT= difucosyl-lacto-N-tetraose, FLNH= fucosyl-lacto-N-hexaose, DFLNH= difucosyl-lacto-N-hexaose, 3'SL= 3'-sialyllactose, 6'SL= 6'-sialyllactose, LSTb= sialyl-lacto-N-tetraose b, LSTc= sialyl-lacto-N-tetraose c, DSLNT= disialyllacto-N-tetraose, DSLNH= disialyl-lacto-N-hexaose, FDSLNH= fucosyl-disialyl-lacto-N-hexaose

**Supplementary Table S3. Eigenvalues and Aggregate Porportions of Variance Explained for the Principal Components in Secretors and Non-Secretors**

| Principal Component | Eigenvalue | Aggregate proportion of variance explained (%) |
|---|---|---|
| **Secretors** | | |
| **PC1** | 4.7 | 25.4 |
| **PC2** | 3.9 | 44.9 |
| **PC3** | 2.2 | 55.8 |
| **Non-Secretors** | | |
| **PC1** | 3.7 | 27.4 |
| **PC2** | 3.3 | 45.2 |
| **PC3** | 2.9 | 56.9 |
| **PC4** | 3.4 | 65.5 |
| **PC5** | 2.3 | 72.0 |

Eigenvalues of the principal components (PCs) that were determined to be positioned above the elbow of the curve n the Scree-plots (Supplementary Figure 1) and therefore included in the analysis; Secretors had three PCs above the elbow of the curve while non-secretors had five. Magnitude of eigenvalue indicates strength of the PC. PC= principal component.

**Supplementary Table S4. Beta coefficients for the Principle Components of Human Milk Oligosaccharides compared between Milk Samples from Mothers Treated with Antidepressants and Anti-inflammatory Drugs Compared to Samples from Unmedicated Mothers, Stratified by Secretor Status**

| Principal Component | Beta coefficient with 95% CI, Adjusted model | |
|---|---|---|
| | Antidepressants vs untreated mothers[a] | Anti-inflammatory drugs vs untreated mothers[b] |
| **Secretors** | | |
| **PC 1** | -0.7 (-1.0, -0.5)* | -0.3 (-0.6, 0.1) |
| **PC 2** | 0.2 (-0.0, 0.5) | -0.0 (-0.4, 0.4) |
| **PC 3** | 0.3 (-0.1, 0,6) | -0.1 (-0.6, 0.4) |
| **Non-secretors** | | |
| **PC 1** | -0.9 (-1.4, -0.5)* | -0.7 (-1.3, 0.1) |
| **PC 2** | 0.1 (-0.4, 0.5) | 0.1 (-0.5, 0.6) |
| **PC 3** | 0.3 (-0.3, 0.9) | 0.0 (-0.6, 0.6) |
| **PC 4** | -0.9 (-1.5, -0.3)* | 0.1 (-0.6, 0.8) |
| **PC 5** | -0.2 (-0.8, 0.4) | -0.0 (-0.8, 0.8) |

*confidence interval not crossing 0.
[a] model adjusted for infant sex, exclusive breastfeeding, child collection age, maternal age, race and ethnicity, maternal body mass index and a composite variable for maternal mood
[b] model adjusted for infant sex, exclusive breastfeeding, child collection age, maternal age, race and ethnicity, maternal body mass index and underlying rheumatic and inflammatory disorders
PC= Principal Component

**Supplementary Table S5. Loading Scores for the Human Milk Oligosaccharides in the Principal Components**

| Secretors[a] | | | | | |
|---|---|---|---|---|---|
| **PC1** | | **PC2** | | **PC3** | |
| LNFP II | 0.83 | LSTc | 0.93 | 3FL | 0.461 |
| LNFP III | 0.75 | DSLNH | 0.93 | FDSLNH | 0.431 |
| DFLNT | 0.75 | 6'SL | 0.92 | LNFP II | 0.381 |
| LNnT | 0.69 | DFLNH | 0.47 | FLNH | 0.296 |
| DSLNT | 0.69 | FLNH | 0.44 | LNH | 0.292 |
| LSTb | 0.63 | LNFP I | 0.33 | DFLNH | -0.30 |

| Non-Secretors[b] | | | | | | | | | |
|---|---|---|---|---|---|---|---|---|---|
| **PC1** | | **PC2** | | **PC3** | | **PC4** | | **PC5** | |
| LNnT | 0.84 | LSTc | 0.86 | LNFP I | 0.90 | LNFP II | 0.81 | DFLac | 0.75 |
| LNFP III | 0.58 | DSLNH | 0.85 | LSTb | 0.84 | FDSLNH | 0.72 | 3'SL | 0.73 |
| DSLNT | 0.44 | 6'SL | 0.76 | LNT | 0.77 | DFLNT | 0.69 | DSLNT | 0.59 |
| FLNH | -0.59 | FLNH | 0.46 | FLNH | 0.29 | LNH | 0.66 | DFLNT | 0.43 |
| 3FL | -0.65 | FDSLNH | 0.43 | DSLNT | 0.28 | LNFP III | 0.48 | LNT | -0.21 |
| DFLNH | -0.76 | LNH | 0.31 | 6'SL | 0.19 | 3FL | -0.34 | LNH | -0.22 |

[a] Loading scores for the human milk oligosaccharides with the greatest contributions for PCs 1-3 in Secretors. A higher magnitude represents a greater contribution to the PC.
[b] Loading scores for the human milk oligosaccharides with the greatest contributions for PCs 1-5 in Non-Secretors. A higher magnitude represents a greater contribution to the PC.
PC= Principal Component, LNFP= lacto-N-fucopentaose, DFLNT= difucosyl-lacto-N-tetraose, LNnT= lacto-N-neotetraose, DSLNT= disialyllacto-N-tetraose, LSTb= sialyl-lacto-N-tetraose b, LSTc= sialyl-lacto-N-tetraose c, DSLNH= disialyl-lacto-N-hexaose, 6’SL= 6’-sialyllactose, DFLNH= difucosyl-lacto-N-hexaose, FLNH= fucosyl-lacto-N-hexaose, 3FL= 3-fucosyllactose, FDSLNH= fucosyl-disialyl-lacto-N-hexaose, LNH= lacto-N-hexaose, LNT= lacto-N-tetraose, DFLNT= difucosyl-lacto-N-tetraose, DFLac= difucosyllactose, 3’SL= 3’-sialyllactose

**Supplementary Table S6. Correlation coefficients between the Human Milk Oligosaccharides and the Principal Components**

| **Secretors[a]** | | | | | |
|---|---|---|---|---|---|
| **PC1** | | **PC2** | | **PC3** | |
| LNFP II | 0.82 | DSLNH | 0.83 | 3FL | 0.472 |
| LNFP III | 0.75 | 6'SL | 0.81 | FDSLNH | 0.419 |
| DFLNT | 0.74 | LSTc | 0.78 | LNFP II | 0.374 |
| LNnT | 0.72 | FLNH | 0.67 | FLNH | 0.277 |
| DSLNT | 0.68 | DFLNH | 0.60 | LNH | 0.276 |
| LSTb | 0.63 | LNH | 0.443 | DFLNH | -0.32 |

| **Non-Secretors[b]** | | | | | | | | | |
|---|---|---|---|---|---|---|---|---|---|
| **PC1** | | **PC2** | | **PC3** | | **PC4** | | **PC5** | |
| LNnT | 0.84 | DSLNH | 0.85 | LSTb | 0.86 | LNFP II | 0.86 | 3'SL | 0.81 |
| LNFP III | 0.71 | LSTc | 0.85 | LNFP I | 0.84 | FDSLNH | 0.75 | DFLac | 0.68 |
| DSLNT | 0.57 | 6'SL | 0.77 | LNT | 0.84 | LNH | 0.70 | DSLNT | 0.63 |
| FLNH | -0.53 | FDSLNH | 0.53 | DSLNT | 0.43 | DFLNT | 0.69 | DFLNT | 0.54 |
| DFLNH | -0.75 | FLNH | 0.52 | 6'SL | 0.32 | LNFP III | 0.62 | DFLNH | -0.29 |
| 3FL | -0.78 | LNT | 0.45 | 3FL | -0.34 | 3FL | -0.54 | FLNH | -0.31 |

[a] Correlation coefficients between the HMOs and the PCs for the HMOs with the greatest contributions for PCs 1-3 in Secretors. A higher magnitude represents a greater correlation with the PC.
[b] Correlation coefficients between the HMOs and the PCs for the HMOs with the greatest contributions for PCs 1-5 in Non-Secretors. A higher magnitude represents a greater correlation with the PC.

HMO= Human Milk Oligosaccharide, PC= Principal Component, LNFP= lacto-N-fucopentaose, DFLNT= difucosyl-lacto-N-tetraose, LNnT= lacto-N-neotetraose, DSLNT= disialyllacto-N-tetraose, LSTb= sialyl-lacto-N-tetraose b, LSTc= disialyl-lacto-N-hexaose, 6’SL= 6’-sialyllactose, LSTc= sialyl-lacto-N-tetraose c, FLNH= fucosyl-lacto-N-hexaose, DFLNH= difucosyl-lacto-N-hexaose, LNH= lacto-N-hexaose, 3FL= 3-fucosyllactose, FDSLNH= fucosyl-disialyl-lacto-N-hexaose, LNT= lacto-N-tetraose, DFLNT= difucosyl-lacto-N-tetraose, DFLac= difucosyllactose, 3’SL= 3’-sialyllactose

**Supplementary Table S7. Component Correlation Matrices for the Principal Component Analyses in Secretors and Non-Secretors**

| **Secretors** | | | | | |
|---|---|---|---|---|---|
| **Component[a]** | **PC1** | **PC2** | **PC3** | | |
| **PC1** | 1 | 0.073 | -0.012 | | |
| **PC2** | 0.073 | 1 | -0.029 | | |
| **PC3** | -0.012 | -0.029 | 1 | | |
| **Non-secretors** | | | | | |
| **Component** | **PC1** | **PC2** | **PC3** | **PC4** | **PC5** |
| **PC1** | 1 | 0.043 | 0.184 | 0.253 | 0.121 |
| **PC2** | 0.043 | 1 | 0.159 | 0.139 | -0.22 |
| **PC3** | 0.184 | 0.159 | 1 | 0.096 | 0.078 |
| **PC4** | 0.253 | 0.139 | 0.096 | 1 | 0.079 |
| **PC5** | 0.121 | -0.22 | 0.078 | 0.079 | 1 |

[a] Correlation coefficients for the correlations between primary components included in the analyses, separately in Secretors and Non-Secretors. Coefficients closer to 1.000 indicate a stronger positive correlation and numbers closer to -1.000 indicate a stronger negative correlation between the principal components. PC = Principal Component

**Supplementary Figure S1. Predicted Effects of Antidepressant and Anti-inflammatory Drug Treatments on the Relative Abundances of Individual Human Milk Oligosaccharides in (A) Secretors and (B) Non-Secretors**

A

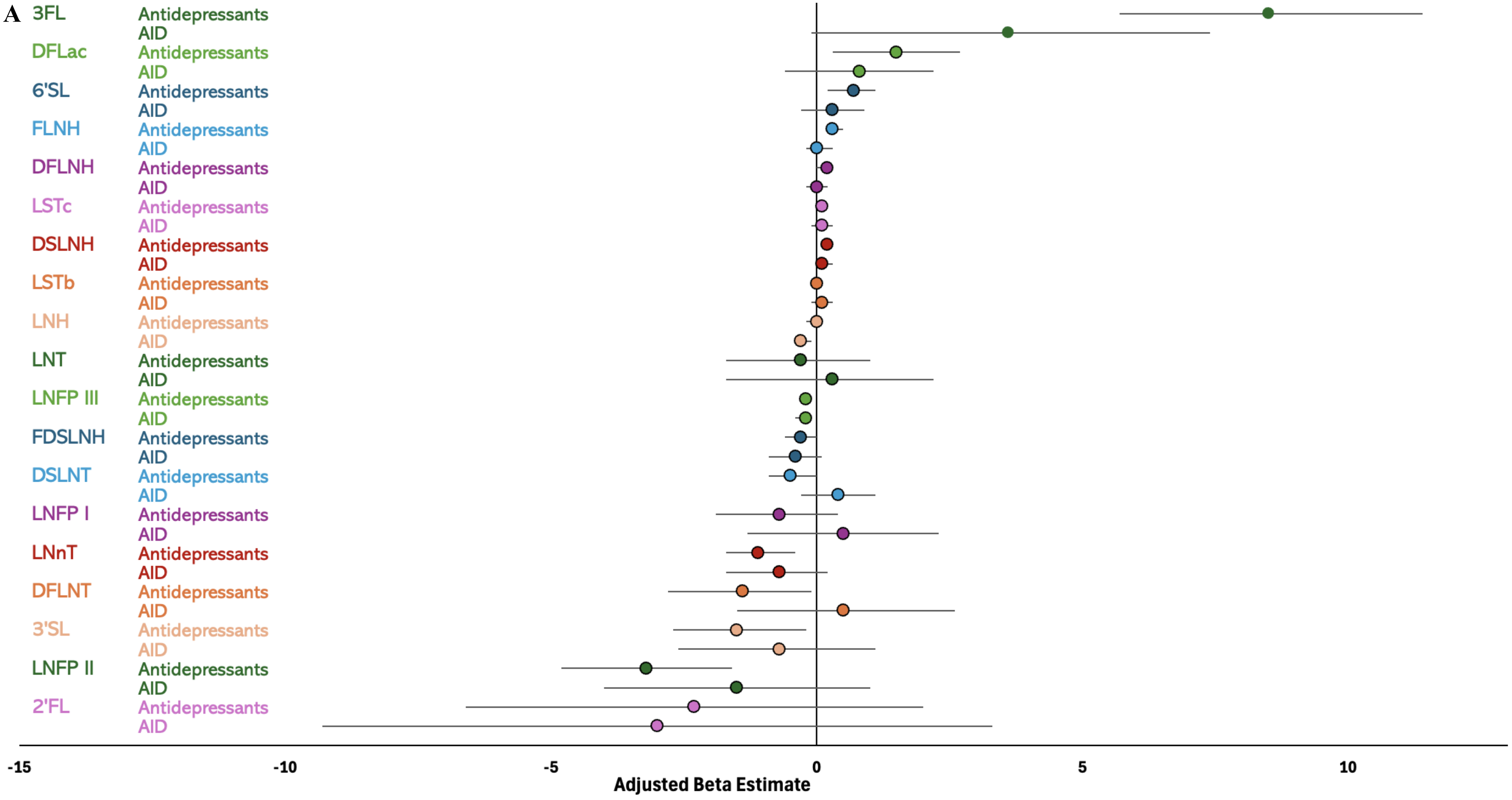

**B**

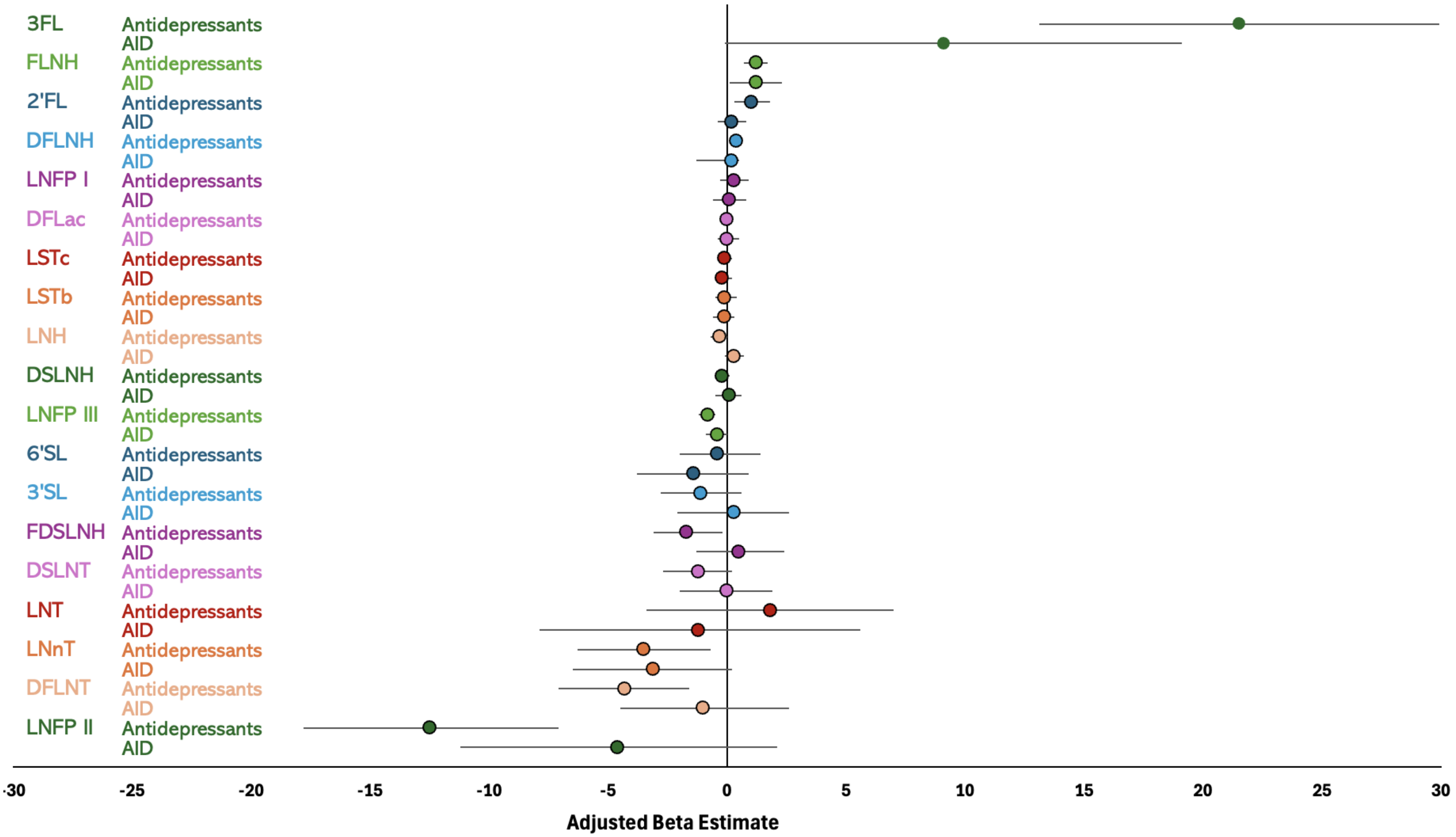


Adjusted beta estimates (markers) with 95% confidence intervals (whiskers) for the predicted effect of antidepressants and anti-inflammatory drugs (AIDs) on the relative abundance of individual HMOs when compared to milk samples from unmedicated mothers, for (A) secretors and (B) non-secretors. Model adjusted for infant sex, exclusive breastfeeding, child collection age, maternal age, smoking, race and ethnicity, maternal body mass index, sample storage time and maternal underlying conditions, which in the comparison of antidepressants was a composite variable constructed of maternal mood disorders and maternal score on Edinburgh

Postnatal Depression Score ≥12, and in the comparison of anti-inflammatory drugs of a current maternal diagnosis of anti-inflammatory or rheumatic disorders.

AID= Anti-inflammatory drugs, 3FL= 3'-fucosyllactose, DFLac= difucosyllactose, 6'SL= 6'-sialyllactose, FLNH= fucosyl-lacto-N-hexaose, DFLNH= difucosyl-lacto-N-hexaose, LSTc= sialyl-LNT b, DSLNH= disialyl-lacto-N-hexaose, LSTb= sialyl-LNT b, LNH= lacto-N-hexaose, LNT= lacto-N-tetraose, LNFP III= lacto-N-fucopentaose III, FDSLNH= fucosyl-disialyl-lacto-N-hexaose, DSLNT= disialyllacto-N-tetraose, LNFP I= lacto-N-fucopentaose I, 2'FL= 2'-fucosyllactose, LNnT= lacto-N-neotetraose, DFLNT= difucosyl-lacto-N-tetraose, 3'SL= 3'-sialyllactose, LNFP II= lacto-N-fucopentaose II

**Supplementary Figure S2. Relative abundances of the individual human milk oligosaccharides**

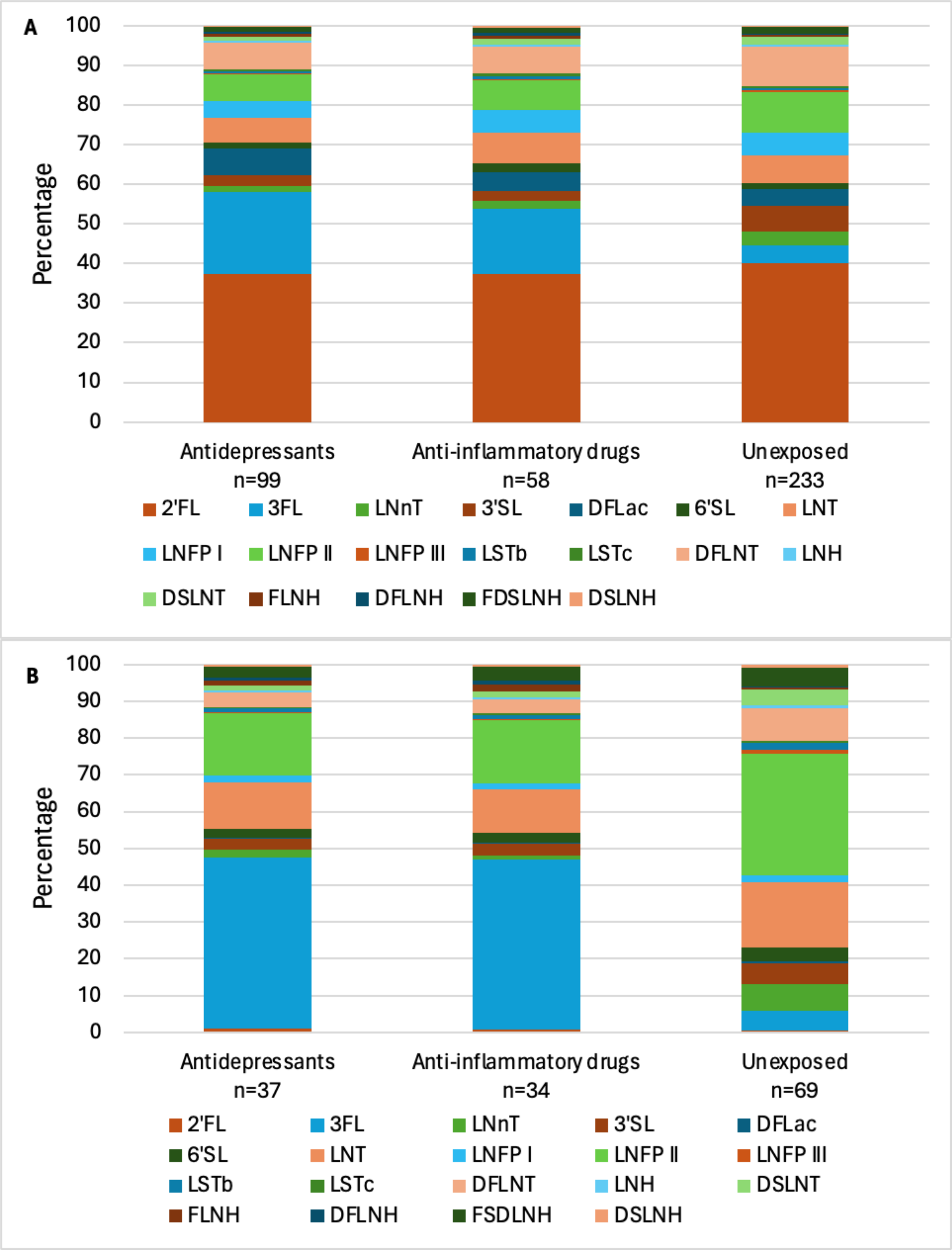

Relative abundances (percentages) of the individual human milk oligosaccharides (HMOs) in milk samples from mothers treated with antidepressants, anti-inflammatory drugs and unexposed mothers in (A) secretors and (B) non-secretors.

2'FL= 2'-fucosyllactose, 3FL= 3'-fucosyllactose, LNnT= lacto-N-neotetraose, 3'SL= 3'-sialyllactose, DFLac= difucosyllactose, 6'SL= 6'-sialyllactose= LNT= lacto-N-tetraose, LNFP I= lacto-N-fucopentaose I, LNFP II= lacto-N-fucopentaose II, LNFP III= lacto-N-fucopentaose III= LSTb= sialyl-lacto-N-tetraose b, LSTc= sialyl-lacto-N-tetraose c, DFLNT= difucosyl-lacto-N-tetraose, LNH= lacto-N-hexaose= DSLNT= disialyl-lacto-N-tetraose, FLNH= fucosyl-lacto-N-hexaose, DFLNH= difucosyl-lacto-N-hexaose, FSDLNH= fucosyl-disialyl-lacto-N-hexaose, DSLNH= disialyl-lacto-N-hexaose

**Supplementary Figure S3. Scree Plots for Eigenvalues of the Principal Components**

**A.**

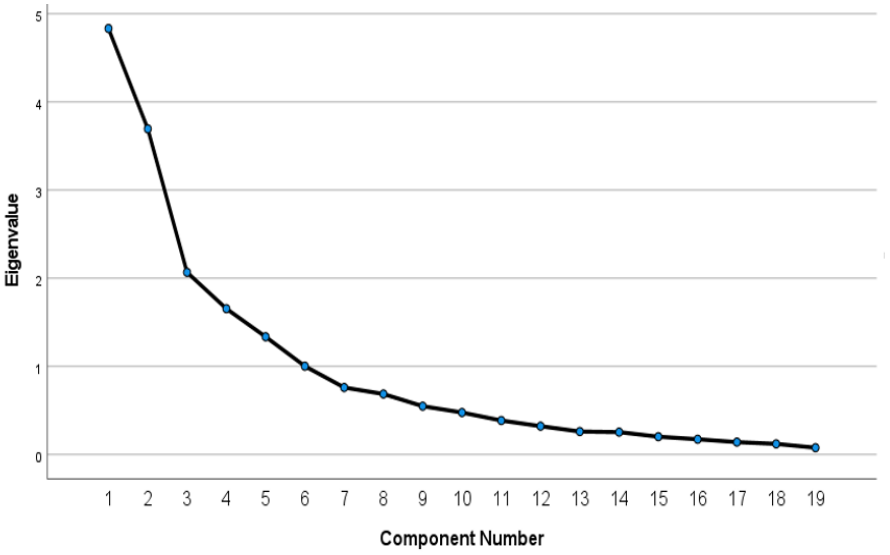


**B.**

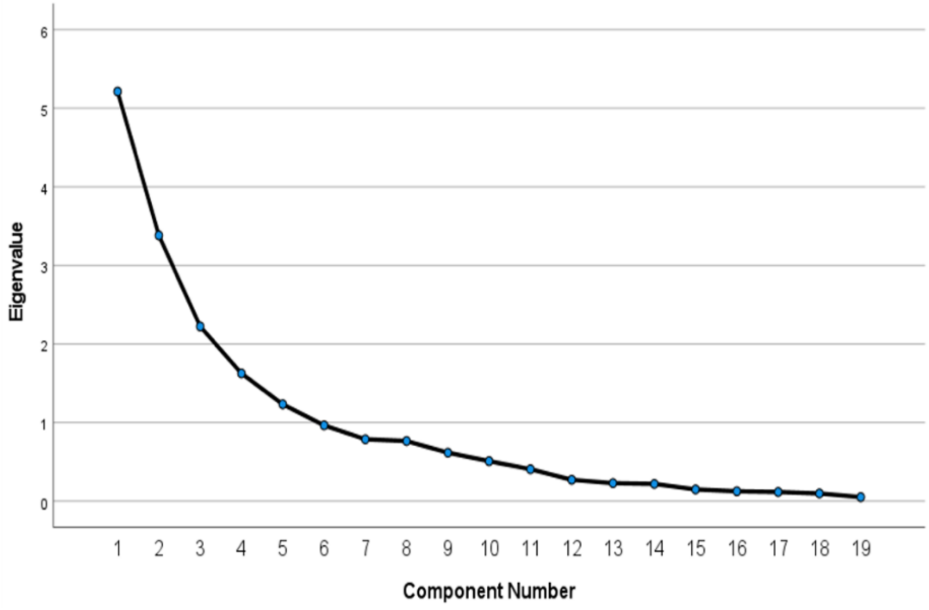


Scree plots for eigenvalues of the principal components for A) Secretors and B) Non-Secretors. The visual elbows of the curves on the scree plots were used as cut-offs for inclusion of principal components in the analyses. Based on this criteria, three principal components were included for secretors and five for non-secretors
